# Carbon-Based Aeronautical Epoxy Nanocomposites: Effectiveness of Atomic Force Microscopy (AFM) in Investigating the Dispersion of Different Carbonaceous Nanoparticles

**DOI:** 10.3390/polym11050832

**Published:** 2019-05-08

**Authors:** Marialuigia Raimondo, Carlo Naddeo, Luigi Vertuccio, Khalid Lafdi, Andrea Sorrentino, Liberata Guadagno

**Affiliations:** 1 Department of Industrial Engineering, University of Salerno, Via Giovanni Paolo II, 132 – 84084 Fisciano (SA), Italy; cnaddeo@unisa.it (C.N.); lvertuccio@unisa.it (L.V.); lguadagno@unisa.it (L.G.); 2 University of Dayton, 300 College Park, Dayton, OH 45440, USA; klafdi1@udayton.edu; 3 Institute for Polymers, Composites and Biomaterials (IPCB-CNR), via Previati n. 1/E, 23900 Lecco, Italy; andrea.sorrentino@cnr.it

**Keywords:** epoxy nanocomposites, carbonaceous nanofillers, FTIR analysis, Thermosetting resins, exfoliated graphite, surface analysis, Atomic Force Microscopy (AFM)

## Abstract

The capability of Atomic Force Microscopy (AFM) to characterize composite material interfaces can help in the design of new carbon-based nanocomposites by providing useful information on the structure–property relationship. In this paper, the potentiality of AFM is explored to investigate the dispersion and the morphological features of aeronautical epoxy resins loaded with several carbon nanostructured fillers. Fourier Transform Infrared Spectroscopy (FTIR) and thermal investigations of the formulated samples have also been performed. The FTIR results show that, among the examined nanoparticles, exfoliated graphite (EG) with a predominantly two-dimensional (2D) shape favors the hardening process of the epoxy matrix, increasing its reaction rate. As evidenced by the FTIR signal related to the epoxy stretching frequency (907 cm^−1^), the accelerating effect of the EG sample increases as the filler concentration increases. This effect, already observable for curing treatment of 60 min conducted at the low temperature of 125 °C, suggests a very fast opening of epoxy groups at the beginning of the cross-linking process. For all the analyzed samples, the percentage of the curing degree (DC) goes beyond 90%, reaching up to 100% for the EG-based nanocomposites. Besides, the addition of the exfoliated graphite enhances the thermostability of the samples up to about 370 °C, even in the case of very low EG percentages (0.05% by weight).

## 1. Introduction

Atomic force microscopy (AFM) is one of the most powerful characterization techniques for morphological and structural analysis of polymeric nanocomposites thanks to its ability to produce three-dimensional (3D) topographic images with a resolution in the order of nanometers [1,2]. These peculiar characteristics allow for the gathering of information about the morphology by drawing the heights and position of very small features present on the sample surface. The AFM also gives the possibility of carrying out a variety of surface characterizations through the controlled interaction between the microscopic probes and the sample surface. Moreover, the AFM is advantageous because it can be used in ambient conditions with minimum sample preparation; in fact, the sample does not have to be conductive and does not require the metallization process before being submitted to morphological investigation. These capabilities make this technique an extraordinary tool for the direct characterization of a variety of samples having complex morphological organizations. 

In recent years, nanostructured polymeric composites have attracted considerable interest from both the academy and industry. The possibility to manipulate these materials on a nanometric scale allows one to obtain multifunctional materials with completely new properties. For example, the use of carbonaceous nanoparticles such as carbon nanotubes, carbon nanofibers and graphene nanoplatelets as filler in polymeric composites is a well-established and effective strategy aimed at improving mechanical, electrical, and thermal performance [3,4,5,6,7,8,9,10,11,12].

In aeronautical and aerospace fields, the continuous and pressing request for high performance materials combining facile manufacturing process and superior mechanical and thermal properties have stimulated the research in different ways [13]. From this point of view, high performance fiber composites characterized by epoxy-based matrices with very high glass transition temperature and impressive thermal and chemical resistance have demonstrated compelling prospectives [14]. The low density and the good physical resistance make these materials very interesting in structural-functional engineering applications. High performance continuous fiber-reinforced (e.g. carbon fiber, glass fiber) composites with complex geometries can be obtained with a simple manufacturing process and with relative low cost. However, the complete replacement of metallic structure is still impossible due to some limitations related to the polymer matrix properties. The low electrical conductivity and the poor barrier properties are important limits still to be overcome. The addition of functional filler can improve these properties without affecting the processability and the cost of these composites. In this paper, different types of one-dimensional (1D) carbon nanofillers such as multi-walled carbon nanotubes (MWCNTs), as-received and heat-treated carbon nanofibers (CNFs), and two-dimensional (2D) predominant shape exfoliated graphite nanoparticles (EG) have been embedded in the same epoxy matrix for obtaining functional structural materials. In particular, the graphite nanoplatelets embedded in polymeric matrices can replace carbon nanotubes both because they are low-priced and because they offer guarantees in terms of wished performance [15]. A good degree of nanoparticle dispersion in the system generally yields the sought properties in an efficient way.

The main limitation in the widespread use of these nanocomposites is represented by the need to avoid the formation of filler particle agglomerates during the dispersion stage within the polymeric matrix. The poor control on the particle dispersion is also related to the limited knowledge of the particles-matrix interactions. The inadequate control of the interface formation at nanometric level also limits the possibility of thermomechanical improvements allowed by the use of these nanoparticles [16].

In this paper, AFM is used to examine the dispersion state of carbonaceous nanofillers within epoxy resin. Thermogravimetric analysis (TGA) and differential scanning calorimetry (DSC) are used to characterize the thermal behavior of both the epoxy matrix and the corresponding nanocomposites. FTIR, used to monitor the curing process of the samples at different temperatures and filler concentrations, shows that, among the analyzed carbonaceous nanoparticles, EG plays the role of accelerating agent allowing the reduction of the band at 907 cm^−1^ due to the presence of oxirane rings as the nanofiller concentration increases. The results of FTIR investigation are in good agreement with DSC results. In fact, as verified through FTIR analysis, DSC data show that, for conversions up to 20%, the conversion of EG-based nanocomposites is shifted at temperature values lower than 10 °C (i.e. TBD + 6.5%EG sample) compared to the epoxy matrix TBD. In conclusion, AFM morphological investigation, as well as FTIR ad DSC tests, demonstrate that the good levels of carbon nanofiller dispersion into the epoxy mixture not only improve the properties of the nanocomposites, but also affect the curing process.

## 2. Experimental

### 2.1. Materials and Manufacturing of Thermosetting Specimens

**Figure 1:**
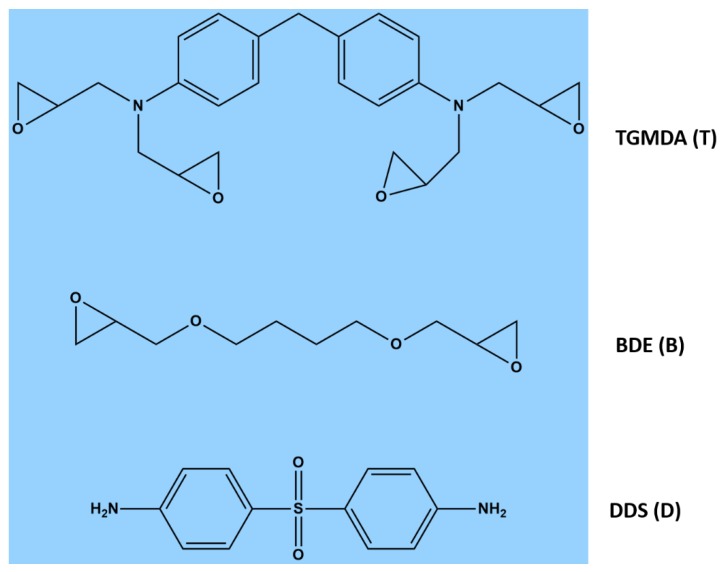
Chemical formulas of tetraglycidyl methylene dianiline (TGMDA) (T), butanedioldiglycidyl ether (BDE) (B), and 4,4-diaminodiphenyl sulfone (DDS) (D).

In this work, the unfilled formulation TBD contains the epoxy precursor T, namely tetraglycidyl methylene dianiline (TGMDA), the reactive diluent B, namely 1-4 butanedioldiglycidyl ether (BDE) mixed at 80% and 20% by weight, respectively and the hardener agent D, namely 4,4-diaminodiphenyl sulfone (DDS), added to the TB epoxy mixture in stoichiometric quantity. The diluent BDE (B) was used to adjust the viscosity of the mixture, in order to facilitate the nanoparticle dispersion in the resulting thermosetting composites, and better control the degassing process during the curing stage [17,18]. In addition, BDE (B) was found to be particularly effective in increasing the degree of curing of nanocharged epoxy resins [19]. In Figure 1, the chemical formulas of all the components (purchased from Sigma-Aldrich) are displayed. 

Three different carbon nanostructured particles (MWCNTs, CNFs, EG) were used as filler in the epoxy matrix. In particular, MWCNTs (3100 Grade) were acquired from Nanocyl S.A. and analyzed by transmission electron microscopy (TEM). The outer diameter and the length of these MWCNTs range from 10 to 30 and from 100 to 1000 nm, respectively. Brunauer–Emmett–Teller method has allowed us to determine the specific surface area of MWCNTs whose value is about 250–300 m^2^/g. The thermogravimetric analysis showed a carbon purity greater than 95%. 

CNFs (PR25XTPS1100, Pyrograf III—Applied Sciences Inc., Cedarville, OH, USA) were produced by pyrolytically stripping at 1100 °C (here named CNFs as-received) and subsequently heat-treated at 2500 °C (here named CNFs heat-treated) to improve the electrical properties [3]. 

The exfoliated graphite (EG) was prepared by mixing a sulphonitrating mixture with natural graphite (Asbury graphite grade 3759, Asbury Carbons, NJ), according to the procedure described in our previous paper [6]. EG particles are characterized by an average diameter of 500 µm and a degree of exfoliated phase equal to 56% [6]. 

TGMDA (T), BDE (B) and DDS (D), giving the TBD sample, were mixed at 120 °C and then added with 1D and 2D carbonaceous nanofillers at different load levels from 0.05 to 6.5% by weight, depending on the nanoparticle type. The mixtures were sonicated (Hielscher model UP200S) for 20 min at 24 kHz and 200W. Process conditions were chosen by considering the mechanical and electrical conductivity of the resulting composites [3,5,6].

The curing process was carried out in two isothermal stages: a first stage at 125 °C for 1 h followed by a second stage at higher temperatures reaching up to 180 and 200 °C for 3 h. This procedure contemplates, for the first curing step, times and temperatures lower than those required for the second step. It corresponds to the common industrial conditions aimed at ensuring an optimal impregnation of the carbon fibers at lower temperature before the resin solidification at higher temperature takes place. 

The nanocomposites were coded as TBD + X(nanofiller), where X is the nanofiller percentage.

### 2.2. Characterizations

Infrared spectroscopy (FTIR) tests were carried out with a Bruker Vertex 70 FTIR-spectrophotometer in the range of 4000-400 cm^−1^, with a resolution of 2 cm^−1^ (32 scans collected). The infrared spectra were obtained in absorbance. 

Thermogravimetric analysis (TGA) was carried out with a Mettler TGA/SDTA 851 thermal analyzer, in the range 25–1000 °C at a heating rate of 10 °C min^−1^ under air flow. 

Differential thermal analysis (DSC) was carried out with a Mettler DSC 822 differential scanning calorimeter in a flowing nitrogen atmosphere, in the temperature range of 30–300 °C with a scan rate of 10 °C min^−1^. 

Morphological investigation by atomic force microscopy (AFM) was carried out with a Dimension 3100 coupled with a Bruker NanoScope V multimode AFM (Digital Instruments, Santa Barbara, CA, USA). The image acquisitions were performed in tapping mode and ambient atmosphere. Commercial probe tips with nominal spring constants of 20–100 N m^−1^, resonance frequencies of 200–400 kHz, and tip radius of 5–10 nm were used. The images were analyzed using the Bruker software Nanoscope Analysis 1.80 (Build R1.126200). Sample slices were cut from samples by a sledge microtome and etched with potassium permanganate in a solution mixture of 95 mL sulfuric acid (95%–97%) and 48 mL orthophosphoric acid (85%) for 36 h. The samples were then washed in water and hydrogen peroxide and dried under vacuum for 5 days. 

**Figure 2:**
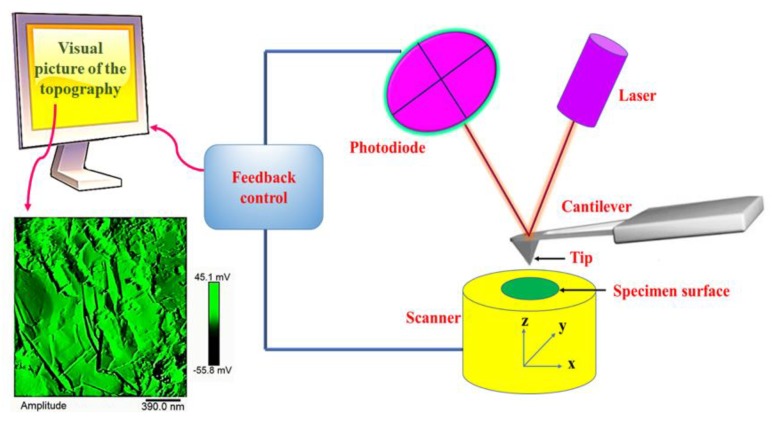
Schematic drawing of an atomic force microscope (AFM): a sharp tip scans over the specimen surface, the piezoelectric scanner moves the specimen (or the probe tip) in xyz directions. A feedback-loop controls the distance between tip and specimen, which is evaluated by a computer and results in a visual picture of the sample topography.

Concerning the working principle of Atomic Force Microscopy (AFM), the AFM topographic images are achieved with a very sharp probe on the whole surface of the sample. The attractive force that develops when the tip approaches the surface, determines the deflection of the cantilever. The changes in sample topography produce a cantilever deflection towards or away from the surface. This deflection is detected by a laser beam, whose reflection moves on the surface of the position-sensitive photodiode. As the cantilever moves through its interaction with the surface, the power of the photodiode signal also changes. Thus, the photodiode registers the cantilever deflection and the subsequent change in direction of the reflected beam when an AFM tip crosses a lifted-up surface feature. The visualization of the topographic image can be obtained by scanning the cantilever on a portion of interest of the sample surface. The cantilever deflection, monitored by the position sensing photodiode, is strongly affected by the higher and lower morphological features appearing on the investigated sample parts. The AFM can create a well-defined topographic map of the surface characteristics through a feedback loop aimed at controlling the height of the tip above the surface [20,21,22]. Figure 2 shows the schematic setup of a typical atomic force microscope AFM.

The AFM measurements can be operated in three different modes: non-contact, contact and tapping mode. AFM tapping mode has been used to characterize polymeric surfaces with nanoscale resolution and minimum sample damage [23,24].

SEM micrographs were obtained using a field emission scanning electron microscope (FESEM, mod. LEO 1525, Carl Zeiss SMT AG, Oberkochen, Germany).

Transmission electron microscopy (TEM) was carried out on a Philips CM-20 model equipped with a 200 kV accelerating voltage and a high brightness LaB6 gun for high coherence and a small probe.

## 3. Results and Discussion

**Figure 3:**
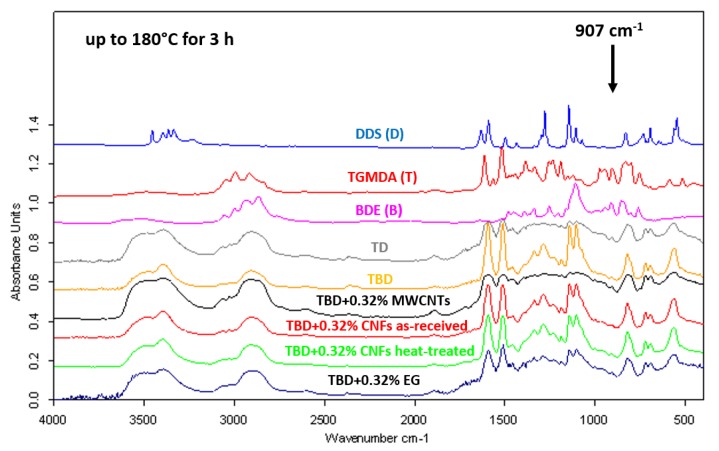
FTIR spectra of neat and nanocomposite systems cured up to 180 °C for 3 h.

FTIR spectra of neat and nanocomposite systems (0.32% by wt of nanofiller) cured up to 180 °C for 3 h are shown in Figure 3. As evident from the figure, the absorbing bands of the O–H stretch at 3200–3650 cm^−1^ increase as the reaction proceeds. At the same time, the curing reaction produces the disappearing of both asymmetric ring-stretching band of the epoxy ring (907 cm^−1^) and the N–H stretching vibration bands of DDS (D) (3060–3500 cm^−1^). When the curing process is complete, only very little C–O–C band of the epoxy ring and N–H stretch bands are detectable. This means that, substantially, the totality of the amine and epoxy groups react giving rise to the crosslinked network. Besides, FTIR analysis confirms the effectiveness of the BDE (B) in determining an improvement in the curing degree of epoxy nanocomposites [19].

In this regard, Figure 3 clearly shows that the rate of epoxy/amine polymerization of TBD unfilled sample (with diluent), estimated by the progressive decrease of the epoxide band intensity, is higher than that of TD unfilled sample (without diluent). 

**Figure 4:**
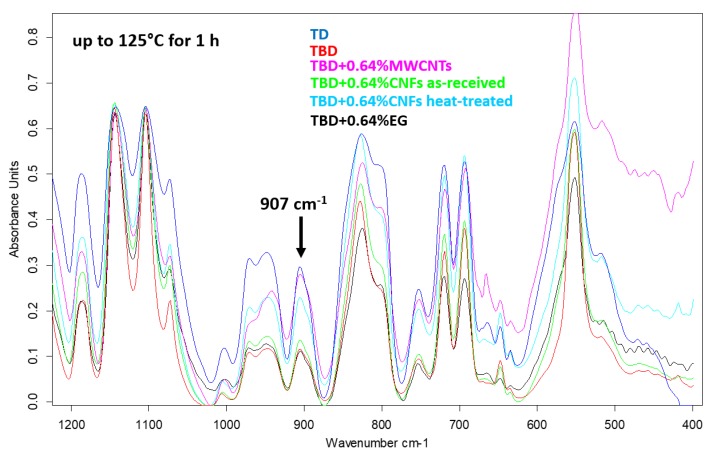
FTIR spectra of neat and nanocomposite systems cured up to 125 °C for 1 h.

The decrease of the epoxide group content of the TBD sample with respect to the TD sample is strongly evident also after an intermediate step of the curing process, at the temperature of 125 °C for 1 h (see Figure 4). Among the different nanofillers, EG nanoparticles speed up the curing process of the epoxy resin with consequent decrease of the epoxy stretching frequency (907 cm^−1^) as it is evident from the comparison between epoxy samples filled with 0.64% by wt of MWCNTs, CNFs as-received, CNFs heat-treated and EG nanoparticles cured up to 125 °C for 1 h (see Figure 4).

**Figure 5:**
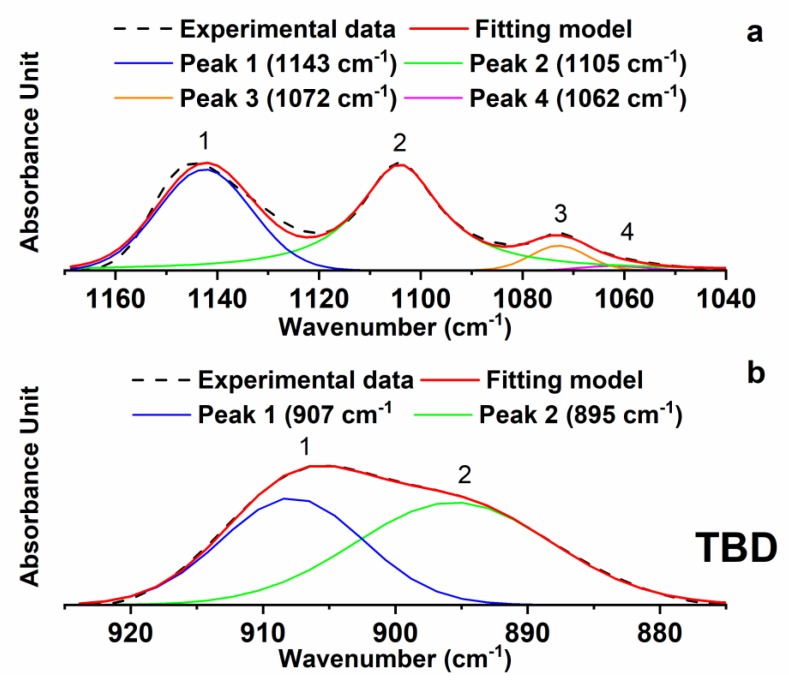
Deconvolution of the FTIR spectrum of the sample TBD collected in the region of the epoxy group (**b**) and the SO_2_ stretching signals (**a**).

The effect of the reactive diluent BDE (B) on the viscosity of the epoxy matrix has been studied in previous papers [17,18]. As stated above, when it is mixed with the TGMDA (T) epoxy precursor, it decreases the viscosity of the epoxy matrix. The decrease of the amount of the epoxy groups of the TBD sample with respect to the TD sample is most likely due to the reduction of the viscosity of the epoxy matrix. To better evaluate the curing degree of the developed nanocomposites, the FTIR bands characteristic of the epoxy group (in the range 860 cm^−1^ and 920 cm^−1^) and the strong asymmetric and symmetric SO_2_ stretching peaks at 1143 cm^−1^ and 1105 cm^−1^ (in the range 1000 cm^−1^ and 1200 cm^−1^) were decomposed into their different components using a complex fitting in which a Lorentzian and Gaussian contribution were considered in the form:

(1) $$f(x)=\left(1-L\right)H\;\exp-\left[{\left(\frac{x-x_{0}}{w}\right)}^{2}\left(4\ln2\right)\right]+L\frac{H}{4{\left(\frac{x-x_{0}}{w}\right)}^{2}+1}$$

where x_0_ is the peak position, H is the height, w is the width at half-height and L is the Lorentzian component. The results of this deconvolution procedure for the sample TBD in the above-mentioned ranges of wavenumbers is shown in Figure 5.

It is worth noting that the curing process does not influence the intensity of the signals corresponding to the strong asymmetric and symmetric SO_2_ stretching peaks at 1143 cm^−1^ and 1105 cm^−1^. This allows us to use one of these bands as an internal reference to analyze the change in the intensity of the band due to the epoxy ring at 907 cm^−1^, which is diagnostic for the evaluation of the extent of the curing reactions and therefore it is directly related to the curing degree of the sample. The described deconvolution procedure, shown for the sample TBD, has been applied to all the formulated nanocomposites. The ratio between the areas of band at 907 cm^−1^ and at 1105 cm^−1^ (A (907)/A (1105)) and the ratio of the intensities (in terms of height of the signals) of band at 907 cm^−1^ and at 1105 cm^−1^ (I (907)/I (1105)) are shown in Table 1 for all the formulated nanocomposites. 

From data reported in Table 1, it is evident that the presence of the reactive diluent BDE (B) determines a strong increase in the curing degree of the matrix. In fact, both the ratios, between the areas (A (907)/A (1105)) and the intensities (I (907)/I (1105)) of the peaks show a relevant reduction going from 0.149 to 0.055 for the ratio between the areas and from 0.413 to 0.126 for the ratio between the intensities. All the formulated nanocomposites, for the same curing treatment, highlight a higher curing degree with respect to the initial sample TD (without reactive diluent BDE (B)). This proves that the reactive diluent BDE (B) well counterbalances the adverse effect of the viscosity increase, due to the presence of nanofiller, on the curing process of the formulated nanocomposites. From Table 1, it is also evident that the sample EG, in percentage 0.64 wt %, shows a slight increase in the curing degree also with respect to the sample TBD. CNFs heat-treated, MWCNTs seem to cause a slight decrease in the curing degree with respect to the sample TBD; whereas no remarkable changes are observed for the sample containing CNFs (0.64 wt %) as-received, which shows only a very small decrease in the curing degree. In any case, also for MWCNTs, CNFs heat-treated and as-received, for a percentage of nanofiller of 0.64 wt %, the curing degree is consistently higher than the sample TD. 

**Figure 6:**
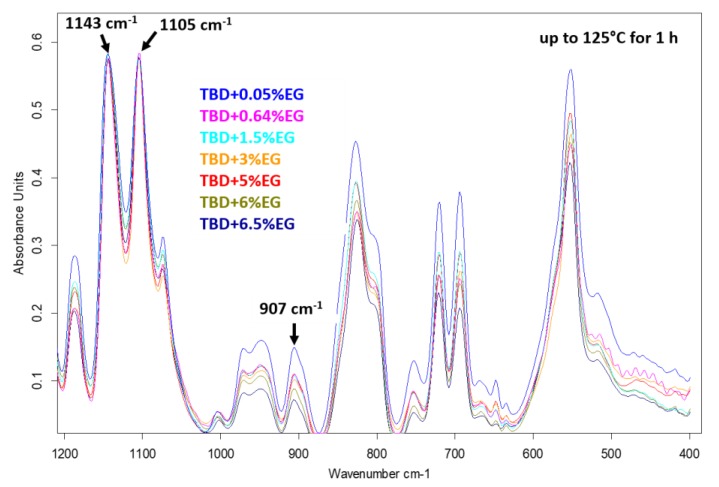
FTIR spectra of EG-based nanocomposites.

For the EG-based samples, an advancing decrease of the epoxy stretching band with increasing concentration is observed (see Figure 6) for the same curing treatment (at 125 °C for 1h). 

**Figure 7:**
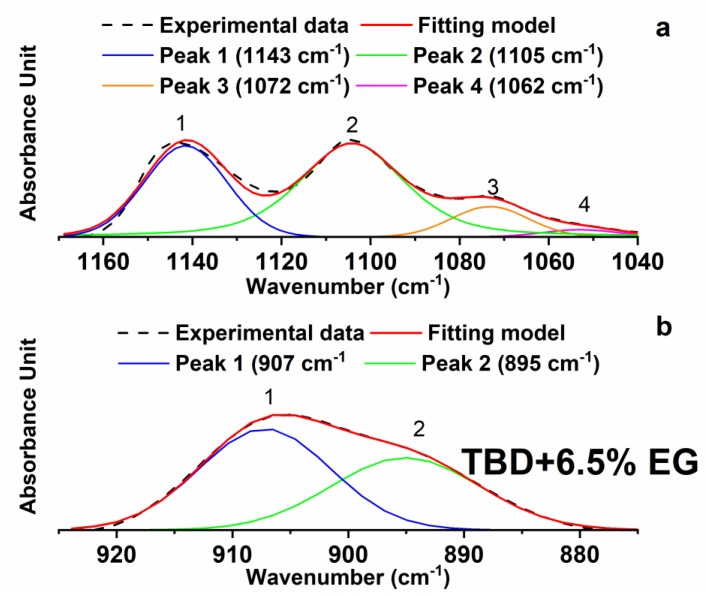
Deconvolution of the FTIR spectrum of the sample TBD + 6.5 %EG collected in the region of the epoxy group (**b**) and the SO_2_ stretching signals (**a**).

It can easily be seen in Figure 6 that, during the curing process, the peaks at 1143 cm^−1^ and 1105 cm^−1^ attributable to the strong asymmetric and symmetric SO_2_ stretchings [25] of the hardener DDS (D) do not change and also the baseline is the same. This allows us a direct visualization of the trend related to the intensity of the epoxy group as the concentration of the nanofiller increases. However, the same deconvolution procedure performed for the FTIR spectra of the samples of Figure 4, has been applied to some of the samples shown in Figure 6. For instance, Figure 7 shows the results obtained for the sample TBD + 6.5%EG. 

The results of the deconvolution procedure obtained for some of these samples (see Table 2) confirm the trend observed in Figure 6. The calculation of the ratios between the areas and the intensities of the peaks at 907 cm^−1^ and 1105 cm^−1^ for these samples shows a reduction of the values by increasing the EG percentage; going from 0.048 (for the percentage of nanofiller 0.64 wt %) to 0.039 (for the percentage of nanofiller of 6.5 wt %). Although the differences in the numbered values of the ratios are not very high, the observed trend seems to confirm a progressive and slight increase in the curing degree with increasing the EG percentage. 

The study of the curing behavior of the nanocomposites during an intermediate stage (in our case after a curing treatment of 125 °C for 1 h) is relevant from an industrial point of view. In fact, in the case of structural panels, for example, Carbon Fiber-Reinforced Panels (CFRPs) impregnated with nanofilled resins, a two-steps curing cycle is frequently adopted. This allows, during the first step at lower temperature, a better impregnation of the carbon fabric. Together with the study of the resin behavior after the first step of the curing cycle (1 h at 125 °C), it is also important the evaluation of the curing degree of the formulated nanocomposite after the complete curing cycle. DC values of EG-based nanocomposites after the complete curing cycle (a first stage at 125 °C for 1 h followed by a second stage at higher temperatures reaching up to 180 and 200 °C for 3 h) are shown in Table 3.

Data shown in Table 3 highlight that the curing degree of the EG-based nanocomposites is higher than 95% for all the investigated filler percentages, proving a good potentiality for eventual applications.

**Figure 8:**
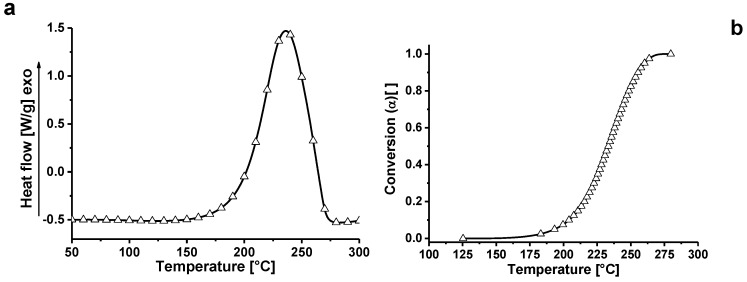
Non-isothermal DSC enthalpy for the epoxy matrix (**a**); variation of the fractional conversion (α) as a function of temperature for the epoxy matrix (**b**).

In agreement with current literature, the curing degree (DC) has been estimated from DSC tests assuming that the exothermic heat evolved during cure is proportional to the extent of reaction [26,27]. Figure 8a shows the non-isothermal DSC test results carried out on epoxy resin, while Figure 8b shows the variation of the fractional conversion (α) as a function of temperature for the epoxy. A linear baseline has been used to fit the enthalpic curve before and after the exothermic peak. The curing reaction is considered complete when the signal leveled off to the baseline. The fractional conversion (α) as a function of temperature can be calculated as follow:

(2) $$\alpha (T)=\frac{\Delta H{\left(T\right)}_{}}{\Delta H_{TOT}}$$

where Δ*H(T)* is the heat released at temperature *T* and Δ*H_TOT_* is the total heat released during reaction. Both quantities are calculated integrating the area between the exothermic curve and the baseline.

The application of equation 2 allows for evaluation of the evolution of the fractional conversion as a function of the temperature. 

For reactions carried out in isothermal conditions, the curing degree (DC) is most effective for characterizing the sample properties. It can be determined by performing a series of isothermal experiments at various temperatures. Thus, DC can be determined from the following equation:

(3) $$DC=\frac{\Delta H_{iso}\left(T\right)}{\Delta H_{TOT}}\times 100$$

where *ΔH_iso_(T)* is the total heat of curing reaction at temperature T.

The curing degree (DC) for EG-filled samples cured at 200 °C is higher than that observed for unfilled epoxy resin, reaching 100% at the end of the test (see Table 3). In any case, the DC values are higher than 90% for all the formulations cured at 200 °C. 

**Figure 9:**
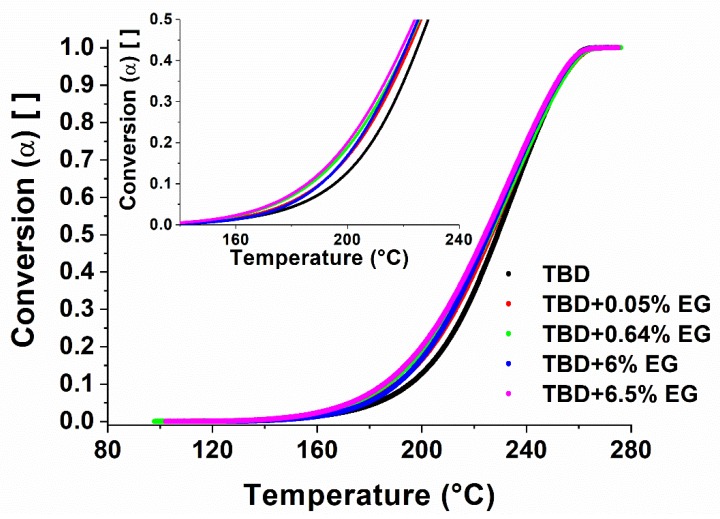
Conversion vs temperature for EG-based epoxy nanocomposites.

As verified in FTIR analysis of Figure 6, the DSC analysis (see Figure 9) show that, for conversions up to 20%, the conversion of the EG systems is shifted of about 10 °C at lower temperatures (i.e. TBD + 6.5%EG) with respect to unfilled epoxy resin TBD. For high degree of conversions, the differences obtained are minimal because the crosslinking phenomena of the matrix are governed exclusively by the curing temperature.

Several authors found that, in the early curing stage, the acceleration effect is due to characteristic of the filler, such as functionalization, specific surface area, presence of catalyst and this effect is evident only for high concentrations [28]. In a previous paper [6], the presence of functional groups was found responsible for the acceleration of the curing reaction. Although the percentage by weight of functional groups present in the filler is low (oxygen content of 0.4 wt %) [6], the acceleration effect becomes relevant for high filler concentrations (6.5 wt %).

**Figure 10:**
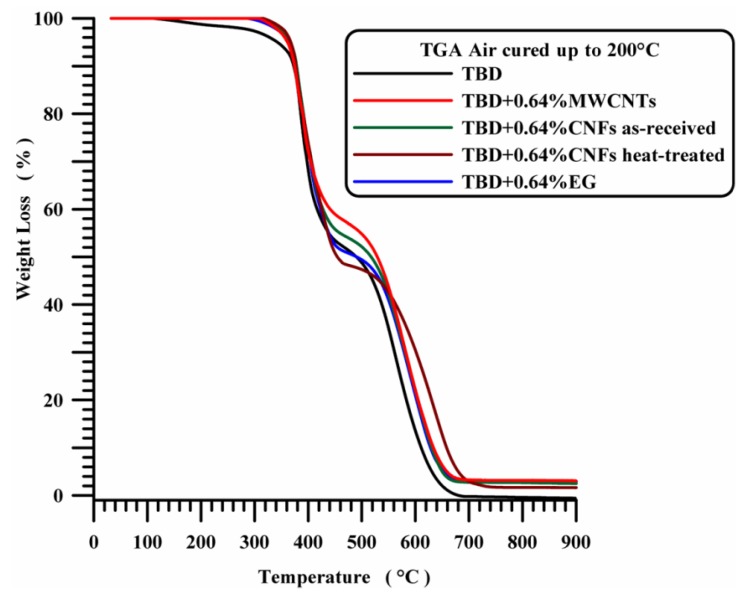
TGA curves in air of neat and nanocomposite systems.

All the nanocomposites show a higher thermal stability compared to unfilled epoxy matrix TBD (see Figure 10). Thermogravimetric analysis of the nanocomposites filled with 0.64 wt % of carbon nanoparticles highlights a stabilizing effect of the nanocomposites in the first degradation stage. 

**Figure 11:**
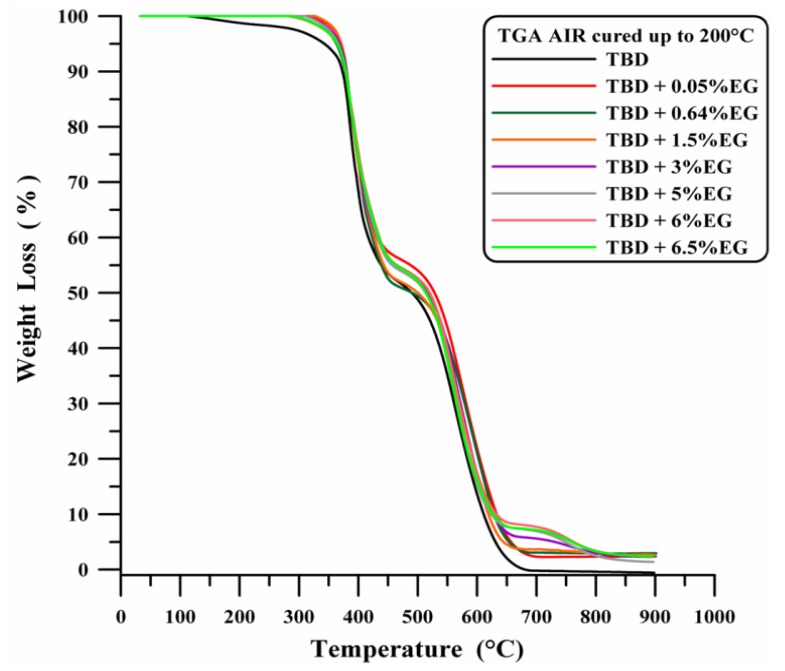
TGA curves in air of neat and EG based nanocomposites.

Figure 11 shows the thermogravimetric curves in air of neat and EG-based nanocomposites, where a considerable increase in the thermal stability up to 370 °C is observed even for a very low percentage of EG (0.05 wt %). EG filled samples demonstrate the advantages that can be obtained from graphene-based materials [29,30,31,32,33].

**Figure 12:**
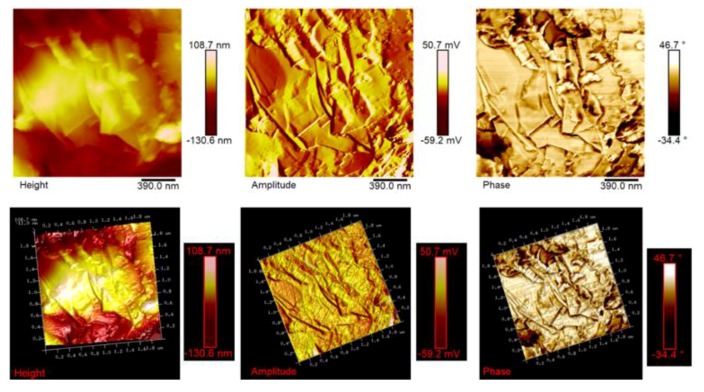
Tapping mode AFM micrographs of the fracture surface of the TBD+3%EG sample (see on the top) and the corresponding 3D profiles (see on the bottom).

**Figure 13:**
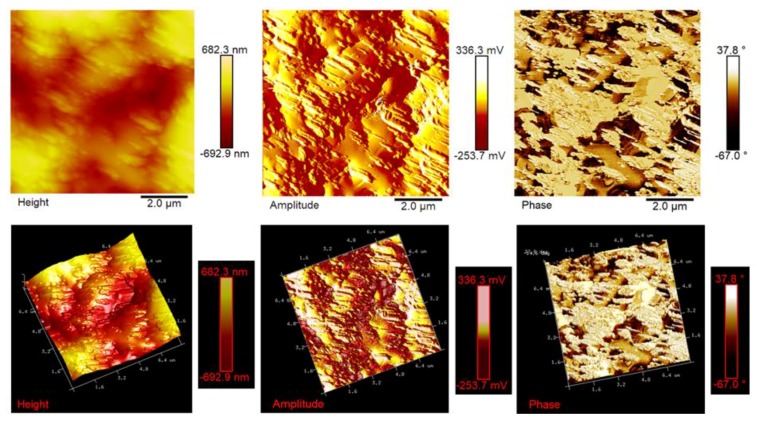
Tapping mode AFM micrographs of the fracture surface of the TBD+0.64%MWCNTs sample (see on the top) and the corresponding 3D profiles (see on the bottom).

**Figure 14:**
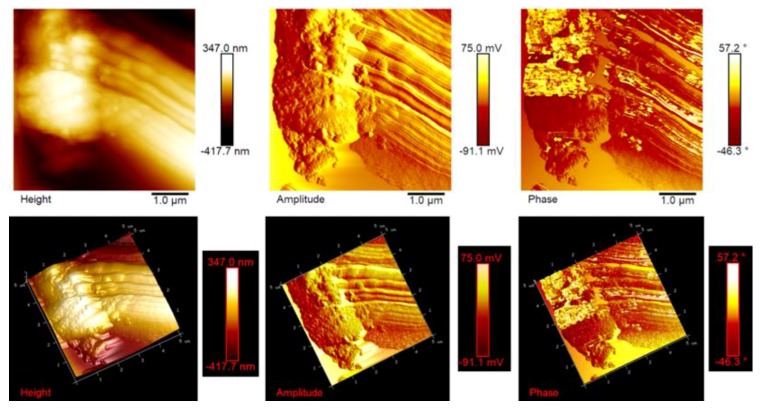
Tapping mode AFM micrographs of the fracture surface of the TBD+1%CNFs heat-treated sample (see on the top) and the corresponding 3D profiles (see on the bottom).

AFM microscopy allows us to analyze the morphology and the properties of the sample surface up to the atomic scale [34,35]. Figure 12, Figure 13 and Figure 14 show the AFM micrographs and the corresponding 3D profiles of the fracture surface of the TBD+3%EG, TBD + 0.64%MWCNTs, and TBD + 1%CNFs heat-treated epoxy samples, respectively. We have chosen to investigate the morphology of these samples because the amount of nanofiller in the mentioned formulations is well above the electric percolation threshold [3,5,6] and therefore represents a good reference to evaluate the effectiveness of the nanofiller dispersion in the resin. In order to obtain reproducible results, the whole surface of the epoxy nanocomposites has been analyzed. In particular, several points of the specimen surface have been scanned to assure the images to be representative. Since the AFM characterization was carried out on etched samples, the carbon nanofillers are well evident and separated from the surrounding epoxy matrix. For each sample, we report three AFM image types: height (or topography) amplitude and phase. The height (or topography) images are obtained by keeping constant the distance between the tip and the sample. These images are very useful as they allow us to estimate both lateral (xy) and height (z) dimensions of the sample features. Nevertheless, the reason why other types of image are usually reported, as in our case, is that such “height profiles” do not always resemble the object under investigation, or more precisely a certain shape may appear very different from what it would have in electron microscopy. This could have the consequence that, for an observer not particularly expert, such images do not easily show the shape of the sample features. The ways in which this happens include shading the image and, most habitually, creating a pseudo-3D image from height data. However, the amplitude (tapping mode) or deflection (contact mode) images represent an effective alternative, since they, being equivalent to a sample slope map, often show the sample shape more readily. It is important, however, to bear in mind that the z-scale in deflection or amplitude is completely meaningless in terms of sample structure. In fact, all that it shows is how the tip folded when it lightly taps the surface of the sample with consequent reduction of the oscillation amplitude. This change in amplitude is used by AFM to trace the surface topography. It is worth noting that the best images are obtained when the amplitude (or deflection) signals are minimized, since the amplitude (or deflection) images are the error signals in AFM technique. The phase images, obtainable in tapping mode, represent a map of how the phase of cantilever oscillation is influenced by its interaction with the surface. In addition to topographic information, the phase image provides insights on the local stiffness due to the direct relation to the material density and elastic modulus [11] thus resulting very sensitive to surface stiffness/softness and adhesion between the tip and surface. In fact, besides to amplitude variations, when a sharp probe approaches near the surface, there are also phase variations determined by a vertical oscillation of the probe near its mechanical resonance frequency. In particular, the changes of the phase signal are detectable when the probe meets areas of the sample with different composition. Phase changes are recorded as luminous and dusky regions in phase images, comparable with color contrasts caused by height changes that are viewable in height images. In general, the phase image is particularly useful in analyzing the surface of mixed (heterogeneous) samples where, in this case, it favors explicit resolution of the various material phases highlighted by a strong contrast of colors between domains because phase imaging allows for chemical mapping of surfaces based on these material differences. AFM technique, through the capture of the height (topography), amplitude and phase data simultaneously, allows surface structure and material domains to be directly compared. 

Atomic force microscopy proved to be an excellent method for assessing the level of dispersion of carbon nanofillers within the polymer matrix for all formulated nanocomposites. The AFM micrographs allow us, in fact, to observe how the different carbon-based nanoparticles are effectively dispersed in the resin. Similar results are obtained for lower concentrations of carbon nanofillers inside the epoxy matrix. They uniformly cover the entire surface of the samples under investigation on which they appear distinctly with the peculiar morphological characteristics, thus also allowing us to irrefutably discriminate between the different type of nanofiller. It is worth noting that the nanoparticles are clearly visible on the surface thanks to the etching procedure, which consumed part of the resin from which they were covered, thus demonstrating the effective design of the nanocomposites that were able to resist the chemical action of the strongly oxidizing solution. In particular, EG-based samples show a very interesting morphological feature (see Figure 12). In this regard, on the fracture surface, we can observe folded graphene sheets that look like a draped fabric.

**Figure 15:**
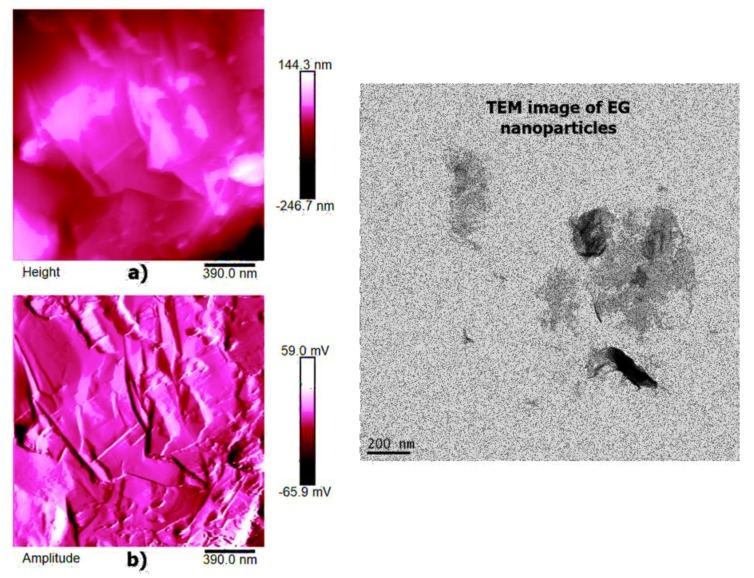
Tapping mode high-resolution AFM micrographs of the fracture surface of the TBD+3%EG sample (see on the left: (**a**) height profile, (**b**) amplitude profile) and the TEM micrograph of EG nanoparticles (see on the right).

**Figure 16:**
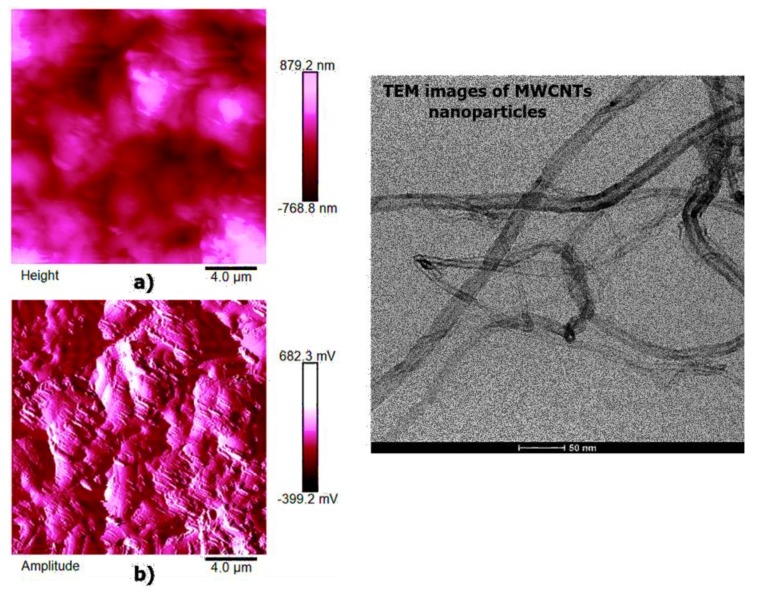
Tapping mode high-resolution AFM micrographs of the fracture surface of the TBD+0.64% MWCNTs sample (see on the left: (**a**) height profile, (**b**) amplitude profile) and the TEM micrograph of MWCNTs nanoparticles (see on the right).

**Figure 17:**
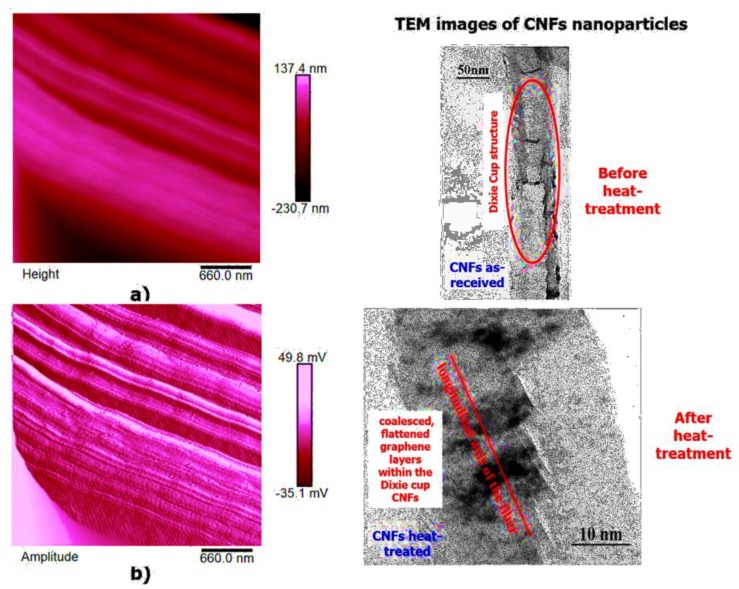
Tapping mode high-resolution AFM micrographs of the fracture surface of the TBD+1%CNFs heat-treated sample (see on the left: (**a**) height profile, (**b**) amplitude profile) and the TEM micrographs of CNFs nanoparticles, before and after heat-treatment (see on the right).

High-resolution AFM micrographs (height and amplitude profiles) of the fracture surface of the TBD + 3%EG, TBD + 0.64%MWCNTs, and TBD+1%CNFs heat-treated epoxy samples, together with the TEM micrographs collected for the individual carbonaceous nanofillers EG, MWCNTs, and CNFs heat-treated are shown in Figure 15, Figure 16, and Figure 17, respectively. It is possible to visualize the morphological peculiarities of the nanoparticles more clearly and directly thanks to the TEM images that provide a valid support in distinguishing their presence inside the epoxy resin. It is worth noting that the TEM images of the CNFs sample (see Figure 17) clearly show the changes in morphology of the carbon nanofibers due to heat-treatment. In fact, the carbon nanofibers, before the thermal treatment are characterized by the peculiar morphology called “Dixie Cup” [3] where the CNFs as-received have an orientation analogous to that of a set of stacked Dixie cups with a hollow core (see red ellipse). At low temperatures, carbon manifests only local molecular ordering. As soon as the temperature of 2500 °C is reached, the warp carbon graphene layers become flattened, giving rise to a straight structure, where a minimum interlayer spacing was reached for the CNFs heat-treated sample. As shown by a TEM micrograph in the Figure 17, the layers within the “Dixie Cup” carbon nanofiber have coalesced following heat-treatment. The rigid and aligned morphology of the heat-treated carbon nanofibers is distinctly visible in the high-resolution AFM micrographs of the fracture surface of the TBD + 1%CNFs heat-treated sample (see Figure 17).

**Figure 18:**
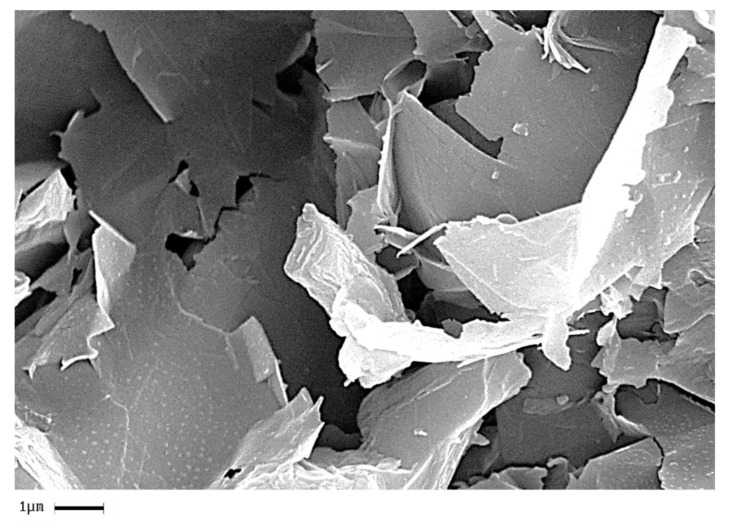
Fracture surface SEM image of the TBD + 3%EG sample.

**Figure 19:**
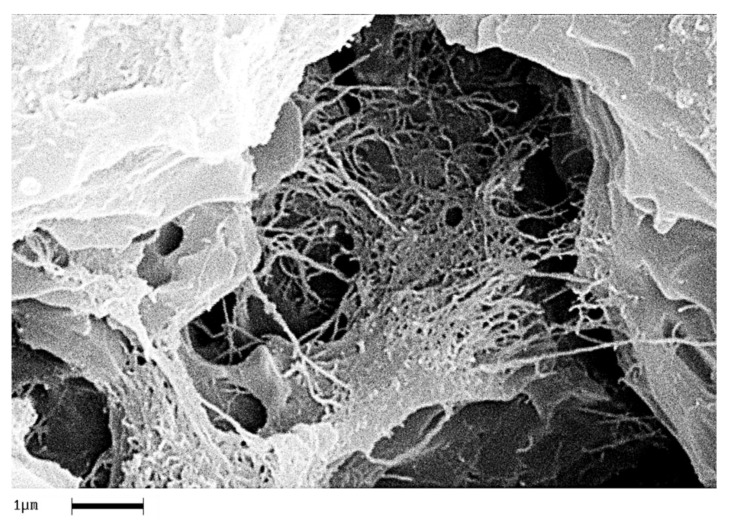
Fracture surface SEM image of the TBD+0.64%MWCNTs sample.

**Figure 20:**
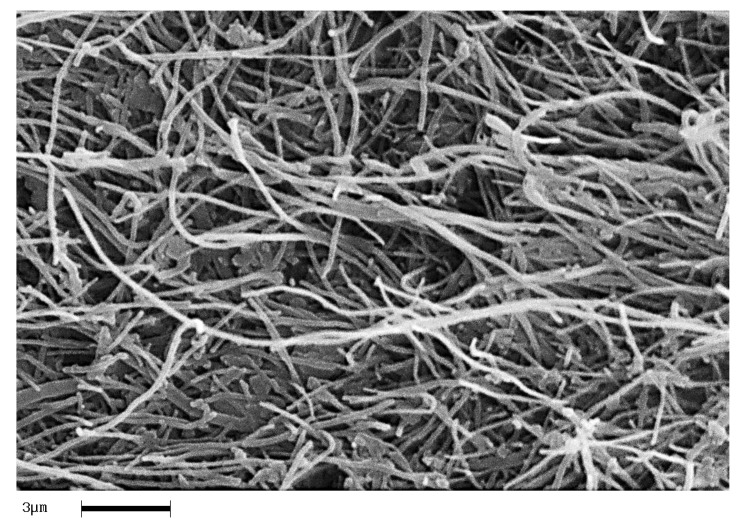
Fracture surface SEM image of the TBD+1%CNFs heat-treated sample.

In order to analyze the homogeneity of the nanofiller dispersion in the polymeric matrix, the epoxy nanocomposites with EG, MWCNTs and CNFs heat-treated were investigated by means of SEM. The analysis was carried out on etched surface fracture of the nanofilled samples. Figure 18, Figure 19 and Figure 20 show the SEM images of nanofilled epoxy resins at the loading rate of 3% by wt of EG, 0.64% by wt of MWCNTs, and 1% by wt of CNFs heat-treated, respectively.

Painstaking observation highlights a homogeneous structure for all the samples, in which the carbonaceous nanofillers are uniformly distributed in the epoxy matrix, thus confirming the morphological results obtained by AFM technique. Besides, it should be pointed out that the structural features of all the nanofillers within the host matrix appear evident, as it is possible to detect by comparing them with the TEM images of the EG, MWCNTs, and CNFs heat-treated nanoparticles shown in the Figure 15, Figure 16 and Figure 17, respectively. 

## 4. Conclusions

The analytical potentiality of AFM technique in examining topographical and nanometric resolution imaging and its effectiveness in investigating the dispersion of different types of nanofillers are explored. Different epoxy nanocomposites have been obtained by adding carbon nanotubes (MWCNTs), carbon nanofibers (CNFs), and exfoliated graphite nanoparticles (EG) to an aeronautic epoxy resin. AFM, TEM, and SEM images highlight good levels of carbon nanofiller dispersion into the epoxy mixture, also allowing us to obtain information about the morphological feature of the nanostructured particles. Thermal and spectroscopic characterizations of these nanocomposites are also considered. FTIR characterization shows that the EG nanoparticles accelerate the curing process of the epoxy resin determining the progressive decrease of the epoxy stretching frequency (907 cm^−1^) with increasing nanofiller concentration. The accelerating effect is in good agreement with DSC results. The curing degree (DC) for the EG-based nanocomposites cured up to 200 °C has been found very high compared to unfilled epoxy resin, reaching up to 100%. A substantial increase in the thermal stability up to 370 °C is also observed even for a very low EG percentage (0.05 wt %).

A consequential result of this investigation lies in the possibility to exploit these promising properties together with the already assessed benefits related to the mechanical and electrical properties. In light of these results, the potential of graphene sheets as promising nanofiller for high-performance nanocomposites in the aeronautical field is well evident.

## Figures and Tables

**Table 1:** Ratios between the areas and the intensities of the peaks at 907 cm^−1^ and 1105 cm^−1^ for the unfilled samples and the nanocomposites containing a percentage of 0.64 wt % of nanofiller.

Sample (cured up to 125 °C for 1 h)	A (907)/A (1105)	I (907)/I (1105)
**TD**	0.149	0.413
**TBD**	0.055	0.126
**TBD+0.64%EG**	0.048	0.119
**TBD+0.64%MWCNTs**	0.100	0.250
**TBD+0.64%CNFs heat-treated**	0.109	0.236
**TBD+0.64%CNFs as-received**	0.063	0.166

**Table 2:** Ratios between the areas and the intensities of the peaks at 907 cm^−1^ and 1105 cm^−1^ for samples containing different percentages of EG nanoparticles.

Sample (cured up to 125 °C for 1 h)	A (907)/A (1105)	I (907)/I (1105)
**TBD+0.64%EG**	0.048	0.119
**TBD+3%EG**	0.042	0.112
**TBD+6.5%EG**	0.039	0.062

**Table 3:** DC values of EG-based nanocomposites.

Epoxy Formulation	Cure Degree (%) Oven cure (200 °C)
**TBD**	91
**TBD+0.05%EG**	100
**TBD+0.64%EG**	96
**TBD+1.5%EG**	100
**TBD+3%EG**	97.5
**TBD+5%EG**	97.3
**TBD+6%EG**	98
